# Supplementary material for: Training large-scale optoelectronic neural networks with dual-neuron optical-artificial learning
Source: Nat Commun. 2023 Nov 4;14:7110. doi: 10.1038/s41467-023-42984-y (PMC10625607; doi:10.1038/s41467-023-42984-y)
Supplement: Supplementary file 1 — Supplementary Information [file 41467_2023_42984_MOESM1_ESM.pdf]

Supplementary Information for Training large-scale optoelectronic neural networks with dual-neuron optical-artificial learning

Xiaoyun Yuan[1,2,3], Yong Wang[1], Zhihao Xu[1,4], Tiankuang Zhou[1,2,3], Lu Fang[1,2,3,*]

[1] Department of Electronic Engineering, Tsinghua University, Beijing, China.

[2] Beijing National Research Center for Information Science and Technology (BNRist), China.

[3] Institute for Brain and Cognitive Science, Tsinghua University (THUIBCS), Beijing, China.

[4] Tsinghua Shenzhen International Graduate School, Shenzhen, China.

[*] Corresponding author. Email: fanglu@tsinghua.edu.cn.

Outline

1. **Supplementary Note S1 and Supplementary Fig. S1 | Dual-neuron layer modeling and optimization.**
2. **Supplementary Note S2 and Supplementary Fig. S2 | Step-by-step procedure of our dual-neuron optical-artificial learning (DANTE) algorithm.**
3. **Supplementary Note S3 and Supplementary Fig. S3| Extending DANTE to other type of ONNs.**
4. **Supplementary Note S4 and Supplementary Table S1| Memory cost and training time of large-scale ONNs.**
5. **Supplementary Note S5 | Implementation Guide of DANTE.**
6. **Supplementary Note S6 and Supplementary Table S2 | Computing performance analysis.**
7. **Supplementary Table S3 | Network scale of existing large-scale ONNs our DANTE.**
8. **Supplementary Fig. S4 | Detailed network structures of Fig. 2.**
9. **Supplementary Fig. S5 | Detailed network structures of Fig. 3 (CIFAR-10 dataset).**
10. **Supplementary Fig. S6 | Detailed network structures of Fig. 3 (ImageNet32 dataset).**
11. **Supplementary Fig. S7 | Optical-artificial fitting error.**
12. **Supplementary Fig. S8 | Convergence analysis of DuAl-Neuron opTical-artificial lEarning (DANTE).**
13. **Supplementary Fig. S9 | DANTE on the custom ONN system.**
14. **Supplementary Fig. S10 | Network structures and learned optical neuron parameters of the ONN system.**

15. Supplementary Movie S1 | Results of our physical ONN system. (MNIST dataset)

16. Supplementary Movie S2 | Results of our physical ONN system. (ImageNet dataset)

Supplementary Notes

Supplementary Note S1 and Supplementary Fig. S1. Dual-neuron layer modeling and optimization. Supplementary Figure S1 illustrates the detailed steps of artificial-neuron layer modeling.

Principle: based on Fourier optics[1,2], in a 2-f system, the optical field of the image plane passing through the lens is Fourier transformed at the output plane (Fig. S1 principle):

$$\mathbf{U}_f(x_i, y_i) = \frac{e^{ik2f}}{i\lambda f} \iint \mathbf{U}_0(x, y) \exp\left[-i\frac{2\pi}{\lambda f}\{xx_i + yy_i\}\right] dxdy, \quad (1)$$

$$\mathbf{U}_f(x_i, y_i) \propto \mathcal{F}_2[\mathbf{U}_0(x, y)] = F_{\mathbf{U}_0}\left[\frac{x}{\lambda f}, \frac{y}{\lambda f}\right]. \quad (2)$$

Step 1: The 4-f system can be considered as performing a single-channel complex-valued convolution combined with a 2D flip operation (Fig. S1, step 1).

$$\mathbf{U}_{f+}(x, y) \propto \mathbf{U}_{f-}(x, y)e^{i\phi_m} = F_{\mathbf{U}_0}\left[\frac{x}{\lambda f}, \frac{y}{\lambda f}\right]e^{i\boldsymbol{\Phi}_m}, \quad (3)$$

$$\mathbf{U}_{4f}(x, y) \propto F_{f+}\left[\frac{x}{\lambda f}, \frac{y}{\lambda f}\right] = \mathcal{F}_2\left[\mathcal{F}_2[\mathbf{U}_0(x, y)]e^{i\boldsymbol{\Phi}_m}\right] \propto \mathbf{U}_0(-x, -y) \otimes \mathbf{PSF}_C. \quad (4)$$

The kernel size is set to match the spatial resolution of the phase mask (2000×2000 in our manuscript). However, the use of large kernels is not prevalent currently[3]. State-of-the-art CNNs, such as VGG net[4] and ResNet[5], instead employ a stack of small spatial convolutions, such as 3×3 and 5×5, to expand their receptive fields. Only a few older network architectures, like AlexNet[6], Inceptions[7], incorporate large spatial convolutions (≥5) as their primary components. This is due to the fact that increasing the kernel size leads to an increase in parameters, resulting in higher training complexity and difficulty. Considering the benefits obtained from this increase, the additional cost incurred is deemed unworthy.

Step 2: Hence, we further decompose the single-channel large-kernel complex-valued convolution operation into a multi-channel complex-valued convolutional operation with small-sized inputs and kernels, serving as the final artificial-neuron layer. This step can significantly reduce the computational cost and memory requirement. The step 2 of the Fig. S2 demonstrates an example of this decomposition. First, the multi-channel input $X^{m \times m \times n_i}$ of the artificial-neuron layer is tiled

into a single-channel complex-valued matrix $X^{\lceil\sqrt{n_i}\rceil(m+2p)\times\lceil\sqrt{n_i}\rceil(m+2p)}$, corresponding to the input of the optical-neuron layer. m is the spatial size of the artificial-neuron layer input, assumed squared for simplicity. p is the padding size, and the gap between the tiled patches is $2p$. n_i is the number of channels of the input. Here, an exemplar tiled optical-neuron layer input is $n_i = 4$ is shown in the subfigure. Second, the multi-channel complex-valued kernel $K^{n_i\times k\times k\times n_o}$ is zero-padded to the same size as the multi-channel input to get $K^{n_i\times m\times m\times n_o}$, and then tiled into a single-channel large kernel $K^{\left(\lceil\sqrt{n_o}\rceil\lceil\sqrt{n_i}\rceil(m+2p)\right)\times\left(\lceil\sqrt{n_o}\rceil\lceil\sqrt{n_i}\rceil(m+2p)\right)}$, to serve as the tiled large-size kernel PSF_C. k is the original kernel size, and n_o is the output channel number of the artificial-neuron layer. As the original kernel is 4D, the tiling consists of two steps. In the first step, the n_i dimension is tiled, generating n_o kernel blocks with sizes of $\lceil\sqrt{n_i}\rceil(m+2p)\times\lceil\sqrt{n_i}\rceil(m+2p)$, represented by the green blocks in the PSF_C. These kernel blocks are then tiled again to form a large-size complex-valued kernel. When convolution is done between the tiled input and tiled kernel, each kernel block will convolve to an entire input, generating an output in the center of the kernel block region in the output plane. These regions will be cropped and used by the following electronic relay (ER) layer.

Step 3: Global artificial-learning is applied to optimize the parameters of the artificial-neuron layers.

Step 4: Local optical-learning is applied to optimize the phase mask. To accurately consider the scale factor λf introduced in the lens-based Fourier transform, we model the optical diffraction step by step to generate the impulse response of the 4-f system.

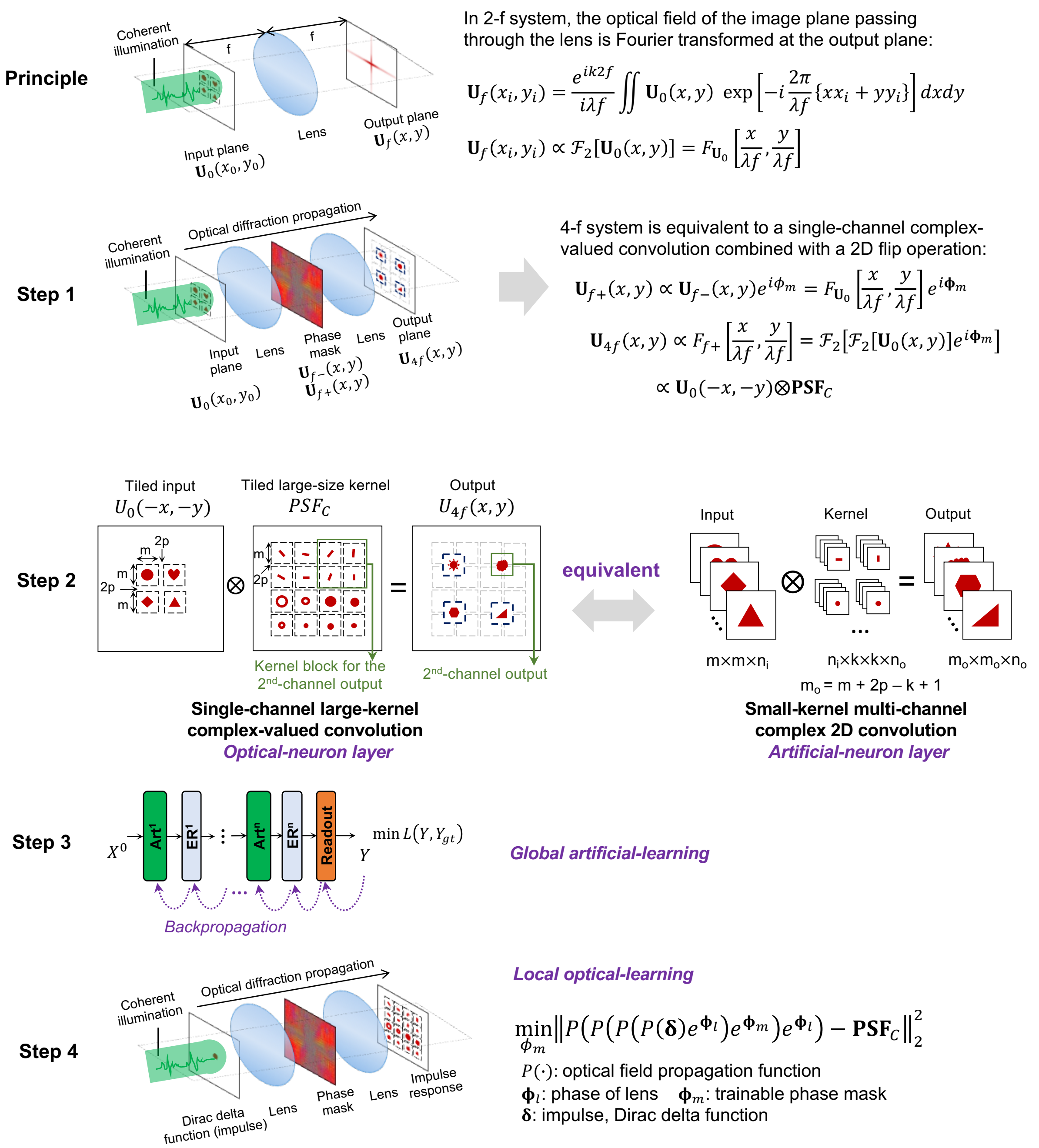

Supplementary Fig. S1 | Artificial-neuron layer modeling and optimization.

Supplementary Note S2 and Supplementary Fig. S2. Step-by-step procedure of our dual-neuron optical-artificial learning (DANTE) algorithm. Supplementary Figure S2 illustrates the step-by-step procedure of DANTE:

Step 1: Initially, the switches connect all the artificial layers to generate a complexed-valued ANN (denoted as ANN_0).

Step 2: Global artificial-learning is conducted to optimize the parameters of all the connected artificial layers.

Step 3: Local optical-learning is carried out on the first un-learned optical-neuron layer, closely matching it to its parallel artificial-neuron layer.

Step 4: If the matching error exceeds the threshold, the switches will connect the learned optical-neuron layers and the artificial-neuron layers with un-learning optical-neuron layers to generate a hybrid network. Global artificial-learning is then performed on the connected artificial-neuron layers to finetune the network and compensate for the introduced matching error. An exemplar hybrid network is illustrated in Supplementary Fig. S1d, if the matching error of second optical modulation exceeds the threshold, the first two optical-neuron layers and the remaining artificial-neuron layers are connected to finetune the network. In our manuscript, the matching error is defined as the decrease in accuracy observed in randomly sampled training data.

Step 5: If the optical-neuron layer can match its parallel artificial-neuron layer well with small enough error, we repeat step 3 on the next un-learned optical layer util all the optical-neuron layers are optimized.

Step 6: Finetune the readout layer (if needed).

For simulation experiments based on the 4-f setup (Fig. 2 and Fig. 3), the artificial-neuron layers can be carefully design so that they can be closely matched by the parallel optical-neuron layers. As a result, both the global artificial-learning stage and local optical-learning stage only need to be conducted once for all the layers. Usually, there are two cases that the ANN has to be finetuned (step 4).

1) Applying our trained optical modulation parameters to a physical system (Fig. 4). The physical assembly error, imperfect laser source, SLM modulation efficiency and lenses introduce

additional errors. Therefore, we have to finetune the ANN to compensate for these errors. In the future, nanofabrication technology can be utilized to solve the deployment error of ONNs.

2) The ONN system has to be updated for new data.

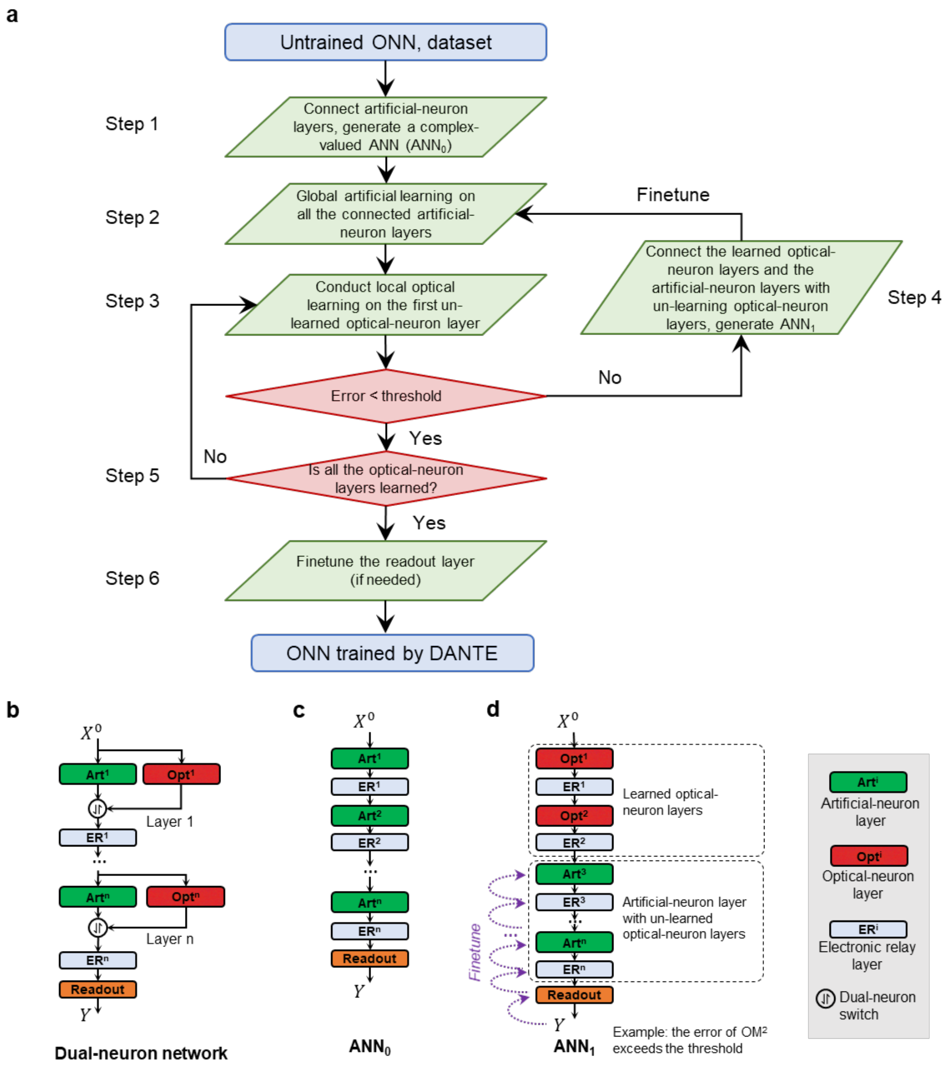

Supplementary Figure S2 | Step-by-step procedure of our dual-neuron optical-artificial learning (DANTE) approach. a, detailed steps of our dual-neuron optical-artificial learning (DANTE) approach. **b,** the dual-neuron network structure. Each layer consists of parallel an artificial-neuron layer (Art in figure) and an optical modulation layer (Opt in figure). The optical-neuron layer accurately models the optical diffraction based on Fourier optics. Meanwhile, the artificial-neuron layer aims to approximate the computationally intensive optical-neuron layer using easily-optimized complex-valued linear computing operations. **c,** the initial artificial neural network generated by connecting all the artificial-neuron layers, denoted as ANN0. **d,** An exemplar hybrid artificial-optical neural network by connecting the first two optical-neuron layers and the remaining artificial-neuron layers. Backpropagation is conducted on the artificial-neuron layers to finetune the network.

Supplementary Note S3 and Supplementary Fig. S3. Extending DANTE to other type of ONNs. Supplementary Figure S3 illustrates an example of extending DANTE to diffractive deep neural networks (D^2NN). The original D^2NN is fully linear. As shown in Supplementary Fig. S3a, we insert electronic relay (ER) layers between these phase masks to introduce nonlinearity for better performance.

Different from the 4-f system, the input-output relation of the D^2NN-like structure can not be perfectly modeled by a complex-valued convolution. Therefore, we divide the whole plane into parallel small blocks. Each block has its own small input region on the input plane, and only corresponds to one single channel of the output (a small region on the output plane). All the blocks are then independently modeled and trained in network learning, and the crosstalk among these blocks can be suppressed by light blockers. After division, the width of the block w_b will be much less than the propagation distance d $(w_b \ll d)$, satisfying the Fresnel approximation. The propagation can be approximated to a 2D FFT operation, and the input-output relation can be modeled by a complex-valued convolution operation (Supplementary Fig. S3b).

$$\mathbf{U}_f(x_i, y_i) = \frac{e^{ikd}}{i\lambda d} \iint \mathbf{U}_0(x, y) \exp\left\{i \frac{k}{2f} [(x_i - x)^2 + (y_i - y)^2]\right\} dxdy \tag{5}$$

$$\mathbf{U}_f(x_i, y_i) \propto \mathcal{F}_2[\mathbf{U}_0(x, y)] \tag{6}$$

$$\mathbf{U}(x, y) \propto \mathcal{F}_2\left[\mathcal{F}_2[\mathbf{U}_0(x, y)] e^{i\mathbf{\Phi}_m}\right] \tag{7}$$

$$\propto \mathbf{U}_0(-x, -y) \otimes \mathbf{PSF}_C \tag{8}$$

Similarly, the phase mask can be optimized by fitting impulse response. As the artificial-neuron layer is modeled under the Fresnel approximation, its optical-neuron layer is not ideally shift-invariant, which will introduce errors in local optical-learning. Therefore, as demonstrated in Supplementary Fig. S3c, we optimize the phase mask to fit multiple impulses (3x3 here) with different spatial shifts, which can enhance the spatial shift-invariant property of the system. $\boldsymbol{\delta}(\Delta x, \Delta y)$ represent the Dirac delta function after shifting $(\Delta x, \Delta y)$ along the x-axis and y-axis direction. $\mathbf{PSF}_C(\Delta x, \Delta y)$ represent the complex-valued kernel after shifting $(\Delta x, \Delta y)$ along the x-axis and y-axis direction.

To validate our extension, we design a D^2NN with two layers, each layer has one phase mask and one electronic relay layer. Similarly, a readout layer is appended to generate the final results. We use the CIFAR-10 dataset to evaluate the network performance. For the first layer, we divide the entire plane into $25(5 \times 5)$ parallel blocks. For the second layer, we divide the entire plane into

36(6 × 6) parallel blocks. In the phase mask optimization of both layers, we optimize 9 (3 × 3) impulse responses with $\Delta x = \{-2, 0, 2\}$ and $\Delta y = \{-2, 0, 2\}$. Existing single-neuron approach achieves an accuracy of 69.7%, cost several days to converge. As a comparison, our DANTE achieves an accuracy of 71.7% within several hours.

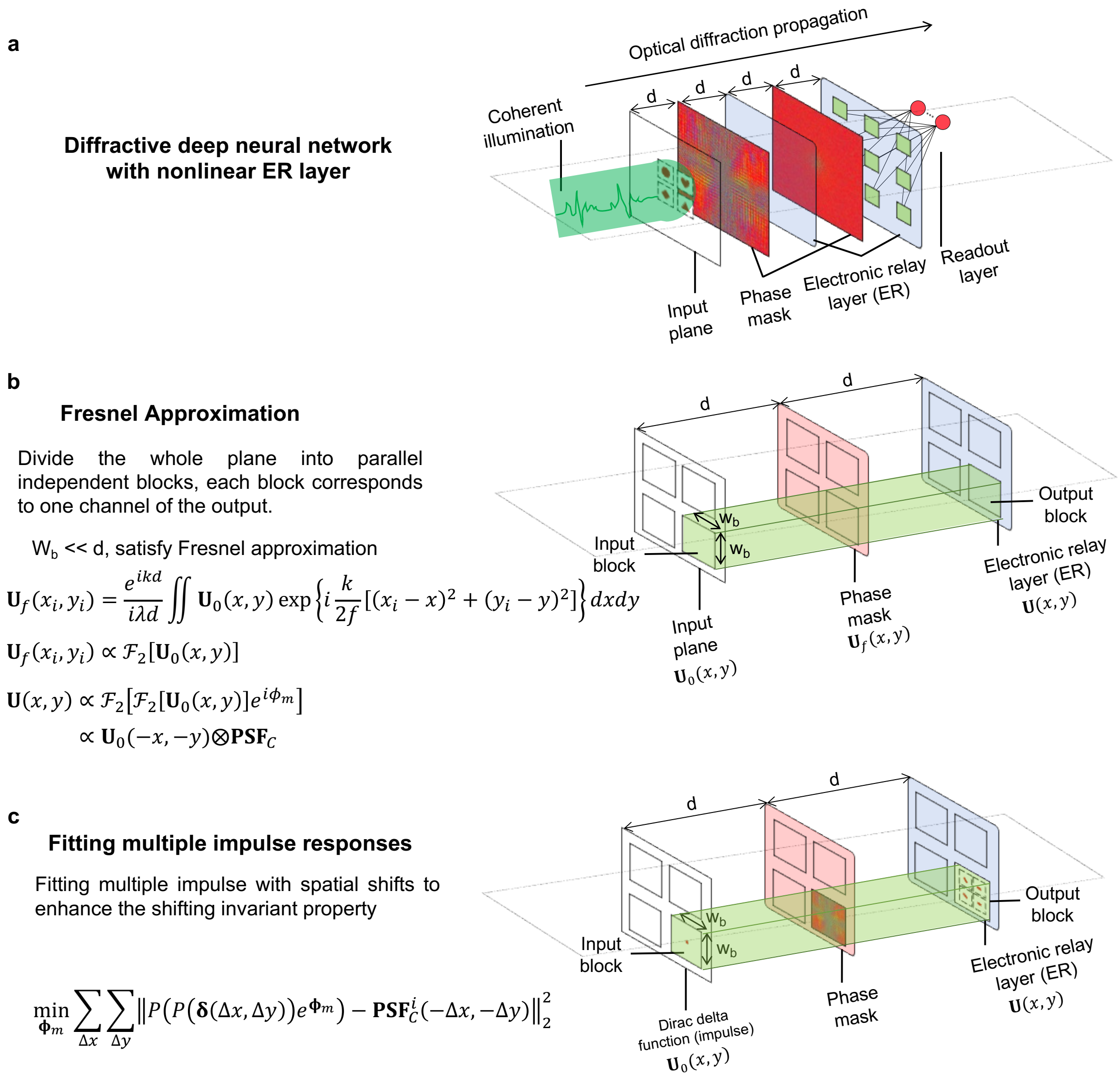

Supplementary Figure S3 | Extending DANTE to other type of ONNs. a, Schematic of our diffractive deep neural networks (D2NN) with nonlinear electronic relay layer. **b,** Divide the entire plane into parallel small blocks to satisfy the Fresnel approximation. **c,** Optimize the phase mask to fit multiple impulses to enhance the spatial shift-invariant property of the system.

Supplementary Note S4 and Supplementary Table S1. Memory cost and training time of large-scale ONNs. The Supplementary Table S1 shows the memory cost and training time per epoch of the large-scale ONNs on the CIFAR-10 dataset in Fig. 3. All the statistics are measured on our Linux server (Nvidia RTX3090 GPU, Intel Xeon Gold 6248R CPU with 96 cores, 256 GB of RAM, and the Ubuntu 18.04.6 LTS operating system) with the PyTorch framework version 1.11.0 (Meta AI).

Existing approach models the entire ONN in the GPU. Since all the phase masks have the same number of trainable parameters, the memory cost and training time per epoch is proportional to the number of trainable phase masks. The red numbers denote the ONNs whose memory cost exceeds 24 GB. At a batch size of 32, only the 3-layer ONN can be trained using only one top-level ordinary GPU (Nvidia RTX 3090, 24 GB VRAM). Even using a small batch size of 4, the memory cost of the 7-layer ONN still exceeds 24 GB. While in DANTE, the memory cost consists of two parts: the global artificial learning (artificial-neuron layer) and the local optical learning (optical-neuron layer). By introducing the artificial-neuron layer in global learning, the memory cost is significantly reduced, and we can use a large batch size of 512 for the 10-layer ONN.

Additional to the memory, the long training time is another issue that makes large-scale ONNs impossible-to-train by previous single-neuron approach. Similar to the memory cost, the training time of a single epoch is also proportional to the number of phase masks. In average, one phase mask requires about 900 s for one epoch on the CIFAR-10 dataset. As a result, training a 7-layer ONN will take approximated 8 hours for one epoch. Since it usually requires tens to hundreds of epochs for a network to converge, training a large-scale ONN on the CIFAR-10 dataset may require several weeks. Conversely, the global artificial-learning stage of DANTE only takes a few seconds for one epoch as the artificial-neuron layer dramatically reduces the computational complexity (9.32 s/epoch for 7-layer ONN). In the local optical-learning stage, the time cost keeps constant as we only need to fit one Dirac delta function for each phase mask. Consequently, it takes about 6500 s to train all the phase masks in the 7-layer ONN, and the entire train processing can be completed within 2 hours.

Supplementary Table S1. Memory cost and training time of large-scale ONNs on CIFAR-10 dataset. #, number; Mem., memory; bs, batch size.

# of layers	3	4	5	6	7	10
# of phase masks	3	4	12	20	30	63
Existing approach (single-neuron)						
Mem. bs = 4 (GB)	2.89	3.16	13.36	20.82	27.42	61.05
Mem. bs = 32 (GB)	23.12	25.28	106.88	166.56	219.36	488.4
Training time / epoch (s)	2585.8	3480.2	10883.1 (~3 h)	18228.3 (~5 h)	28077.0 (~8 h)	58189.9 (~16 h)
DANTE (dual-neuron approach)						
Global artificial Mem. bs = 512 (GB)	2.49	2.89	3.75	4.22	5.08	8.51
Global artificial Training time / epoch (s)	2.55	3.50	7.57	6.60	9.32	18.36
Local optical Mem. bs = 1 (GB)	2.36	2.36	2.36	2.36	2.36	2.36
Local optical Training time / phase mask (s)	218	218	218	218	218	218
Local optical All-layer training time (s)	654	872	2616	4360	6540	13734

Supplementary Note S5. Implementation Guide of DANTE.

Simulation experiment. We have made our code for simulation experiments available, which is implemented using PyTorch. All the necessary information can be found in the source code, covering aspects such as optical diffraction modeling, optical modulation elements, artificial neuron approximation, network structure definition, and the optimization modules for DANTE. More specifically:

1) The optical diffraction modeling is implemented as `class AngSpecProp` in `optical_unit.py`. The forward-pass function of this class is derived based on Fourier optics. The optical elements lens and phase masks are implemented as `class Lens` and `class PhaseMask`. The modulation phase of the `class Lens` is computed from the focal length. The `class PhaseMask` has a trainable variable with a range of the trainable variable is $(-\infty, \infty)$, and a sigmoid function is used to convert the trainable variable to modulation phase $(0,1) \times 2\pi$. These classes are created as extensions of the `torch.nn.Module` class. Their usage closely mirrors that of PyTorch ANN layers like `torch.nn.Linear` and `torch.nn.Conv2d`.
2) The optical-neuron layer (4-f system) is implemented as `class FourierConvComplex` in `optical_layer.py`, which consists of two lens and one phase mask. A function `train_complex()` is implemented to train the modulation phase of the phase mask (Fig. 2abc). The input is a 2D Dirac delta function (impulse), and the label is a large complex-valued kernel obtained by tiling the multi-channel complex-valued kernels. Complex-valued MSE loss and ADAM backpropagation are used to optimize the phase mask.
3) The artificial neuron is implemented as `class ComplexConv2d` in `electric_unit.py`. The complex-valued convolution operation is implemented using 4 real-valued convolution operations in the PyTorch package. The `class ConvBlock` in `electric_unit.py` implements an artificial-neuron layer and a cascaded electronic relay layer. The bias and scale operation in the electronic relay layer is implemented using the `BatchNorm2d layer` in the PyTorch package.
4) The network structure is defined in `electric_network.py` using the above-mentioned classes.
5) The global artificial-learning step is implemented in

`train_electric_cifar10_readout.py`. The local optical-learning step is implemented in `train_electric_optical_kernel.py`. The code can automatically detect the network structure and optimize the optical-neuron layers to fit learned complex-valued kernels of the corresponding artificial-neuron layer. The accuracy testing function is implemented in `test_electric_optical_accuracy.py`. Existing single-neuron learning approach is implemented in `train_end2end_cifar10_readout.py` for comparison.

Physical experiment. The physical ONN system is specifically designed to validate the physical feasibility of DANTE. The optical devices employed are detailed in the Method section. The optical diagram and real system image are provided in Fig. 4. The SLM calibration curve is shown in Supplementary Fig. S9a, and the network structures are demonstrated in Supplementary Fig. S10. The optical components and elements utilized in our system, including the laser, square aperture, lens, polarizer, beam splitter, and the spatial light modulator (SLM), are all widely recognized and commonly used in optical experimental setups. In general, there are two main differences between the modeling in simulation experiment and in the physical experiment:

1) In the simulation experiment, the polarization dimension is not considered for simplicity. In the physical experiment, we have to take the polarization into account, since the SLM is polarization sensitive. Besides, due to the optical activity of liquid crystals, the polarization direction of the optical field will change pixel-wisely after modulation by the SLM. To facilitate modeling of the optical system, we set the SLM-1 to amplitude modulation mode using two polarizers P-1 and P-2. The optical field will then have a fixed polarization direction. Another polarizer P-3 is used to change the polarization direction the same as the fast axis of the SLM-2. Before the experiment, we have to calibrate the SLM-1, and find the relationship between the input SLM pixel value and the modulated optical amplitude (the square root of the optical intensity). The curve can be approximated using a differentiable cosine function and integrated with the neural network model.
2) Due to the imperfect modulation efficiency of the SLM and the assembly error, there will be a zero-order diffraction pattern in the center of the output plane (the sensor plane). The zero-order diffraction pattern is the same as the input optical field. To avoid the interference of the zero-order pattern, we leave the center region blank when tiling the multi-channel kernels into

the single-channel large kernel. After capturing the output optical intensity map using the sensor, the zero-order diffraction pattern can be removed using an intensity-correction mask. More details are presented in Supplementary Fig. S9.

Finally, the step-by-step procedure for utilizing the system is as follows:

3) Upload the network parameters (trained phase masks) to SLM-2.
4) Convert the pixel intensity of the input image or feature map to SLM pixel values using the curve provided in Supplementary Fig. S9a. Then, upload the converted SLM pixel values to SLM-1.
5) Capture the computing results using the sensor, and apply the intensity correction mask (Supplementary Fig. S9b). Crop the pixel that will be utilized in the next layer, and perform average pooling. As the pixel size of SLM is 8 μm, and the pixel size of the CMOS sensor is 3.45 μm, resampling is performed before pooling to match the pixel size.
6) Repeat step 2 and 3, until all the optical modulation layers have been processed.
7) Input the obtained pixel values to the final fully-connected layer to get the final network output.

Supplementary Note S6 and Supplementary Table S2. Computing performance analysis.

In the discussion section of the manuscript, we briefly analyze the computing performance of our ONN system. Here, we do a little bit deeper, discuss the following 2 aspects:

1) Analyze the computing cost of training the artificial-neuron layer and optical-neuron layer from theory. From the result presented in the Fig. 2, Supplementary Note S4 and Table S1, we have shown DANTE can accelerate the training more than 100 times. Here, we discuss why DANTE can achieve such an improvement. In DANTE, we approximate the optical-neuron layer into an artificial-neuron layer, which consists of a complex-valued convolution operation. Assume the convolution operation has an input size $N_i \times N_i$, input channel C_i, kernel size N_k, and output channel C_o. Empirically, they should satisfy $(N_i + N_k) \times \lceil\sqrt{C_i}\rceil \times \lceil\sqrt{C_o}\rceil \leq N_t/2$, where $N_t = 1200$ is the trainable phase mask size, and $(N_i + N_k) \times \lceil\sqrt{C_i}\rceil \times \lceil\sqrt{C_o}\rceil$ approximately equals to the size of the tiled kernel. Exceeding this, the local optical learning will introduce large errors, as the trainable phase mask size can not cover enough frequencies on the Fourier plane. The number of MAD operations (OPs) of this complex-valued convolution is $N_i^2 N_k^2 C_i C_o$. Because $(a + bj)(c + dj) = (ac - bd) + j(ad + bc)$, a complex-number multiply operation needs 4 real-number multiply operation and 2 real-number add operation (6 real-valued OPs). Thus, a complex-valued multiply-add (MAD) operation requires 4 real-number multiply operations and 4 real-number add operations, equals 8 real-valued OPs. The most commonly used kernel size is $N_k = 3$, so the OPs of artificial-neuron layer is $8N_i^2 N_k^2 C_i C_o \approx N_k^2\left(N_i\sqrt{C_i}\sqrt{C_o}\right)^2 = N_k^2\left(\frac{N_t}{2}\right)^2 = 25.92$ MOPs (real-valued). Conversely, in the optical-neuron layer, we model the optical diffraction step by step for solving the size matching problem. The forward process needs 4 FFT, 4 inverse FFT (iFFT), and 7 complex-valued element-wise multiplication. The OPs for FFT/iFFT are around $5N^2 \log_2 N^2$ (real-valued), where N is the spatial size of simulation[8]. Thus, the OPs of an optical-neuron layer are $8 \times 5N^2 \log_2 N^2 + 6 \times 7N^2 = 3677.1$ MOPs (N=2000, real-valued), which is approximately $3677.1/25.9 \approx 140$ times of the artificial-neuron layer. This explains why DANTE can achieve over 100 times acceleration.

2) The artificial-neuron layer TOPs provides a more reliable metric of network performance. As mentioned in the discussion section, different computing techniques may have different definitions for counting the number of operations (OPs). Existing ANNs usually use the FLOPs metric, which

counts number of floating-point calculations at given bit depth, such as FP32, FP16, etc. While currents ONNs works in analog domain, and the arithmetic types and sizes are usually limited. What is more, wave-propagation-based ONNs such as diffractive neural networks are not designed to complete traditional multiplication and addition operations. Miscuglio et al. only compare the inference time between their proposed system and the GPU, without discussing the OPs[9]. Zhou et al. counts the number of optical connections between two surfaces as the OPs, which models the optical diffraction as a fully-connected layer[10]. Therefore, directly comparing the OPs between ANN and ONN is unfair. Here, our DANTE also gives another way to measure the performance of ONN. We have provided the OPs and classification accuracies of our two 10-layer ONNs as well as the ANNs used for comparison in Supplementary Table S2. It can be observed that the total OPs of all the artificial-neuron layers serve as a reliable metric. When the OPs of the artificial-neuron layers in an ONN are similar to the FLOPs of an ANN, their performances tend to be comparable as well. For example, 10-layer ONN vs. VGG11 on the CIFAR-10 dataset, 10-layer ONN vs. VGG16 vs. WRN-1. WRN-k denotes the Wide Residual Networks[11], and k is the widening factor. While network with obviously higher OPs has significantly better performance, like WRN-2. For the VGG networks, since the input dimension of ImageNet-32 (32x32) is different from the original VGG input dimension (224x224), we reduce the size of the fully-connected layers and retrain the network. Hence the VGG FLOPs and performance in our table are different from that online.

Supplementary Table S2. the OPs and classification accuracies of our two 10-layer ONNs and the ANNs used for comparison.

Dataset	CIFAR-10		ImageNet-32			
Network	10-layer ONN	VGG11	10-layer ONN	VGG16	WRN-1	WRN-2
Input dimension	28x28x3	28x28x3	32x32x3	32x32x3	32x32x3	32x32x3
FLOPs (ANN)	/	315.8 M	/	635.3 M	788.6 M	3.14 G
Real-valued OPs (Artificial-neuron layer)	441.0 M	/	808.7 M	/	/	/
# of phase masks	63	/	104	/	/	/
Real-valued OPs (Optical-neuron layer)	231.6 G	/	382.4 G	/	/	/
Accuracy (Top-1/top-5)	89.53%/	89.33%/	44.7%/ 69.0%	41.9%/ 64.4%	42.9%/ 67.5%	50.88%/ 75.24%

Supplementary Table S3. Network scale of existing large-scale ONNs our DANTE.

Supplementary Table S3. Network scale of existing large-scale ONNs our DANTE.

Model	# of layers	# of masks	# of neurons per mask	# of neurons
D-NIN-1[10]	3	7	700x700	3.6 M
Residual-D^{2}NN[12]	30	30	200x200	1.2 M
Ensemble of D^{2}NN[13] N = 30	5	5x30	200x200	0.2 M x 30 = 6 M
Ensemble of D^{2}NN[13] N = 77	5	5x77	200x200	0.2 M x 77 = 15.4 M
5-layer D^{2}NN[14]	5	5	300x300	0.45 M
10-layer D^{2}NN[14]	10	10	300x300	0.9 M
Fourier-space D^{2}NN[15] (cell)	5	5	800x800	3.2 M
Fourier-space D^{2}NN[15] (CIFAR-10)	10	10	160x160	0.26 M
Amplitude-only Fourier neural network[9]	1	16	832x832	11.08 M
D^{2}NN at Visible Wavelengths[16]	5	5	1000x1000	5.0 M
Programmable D^{2}NN[17]	5	5	8x8	320
Multiscale diffractive U-Net[18]	11	11	128x128 (max) layer different	0.12 M
Ours (10-layer ImageNet)	10	104	1200x1200	149.8 M

Supplementary Figures

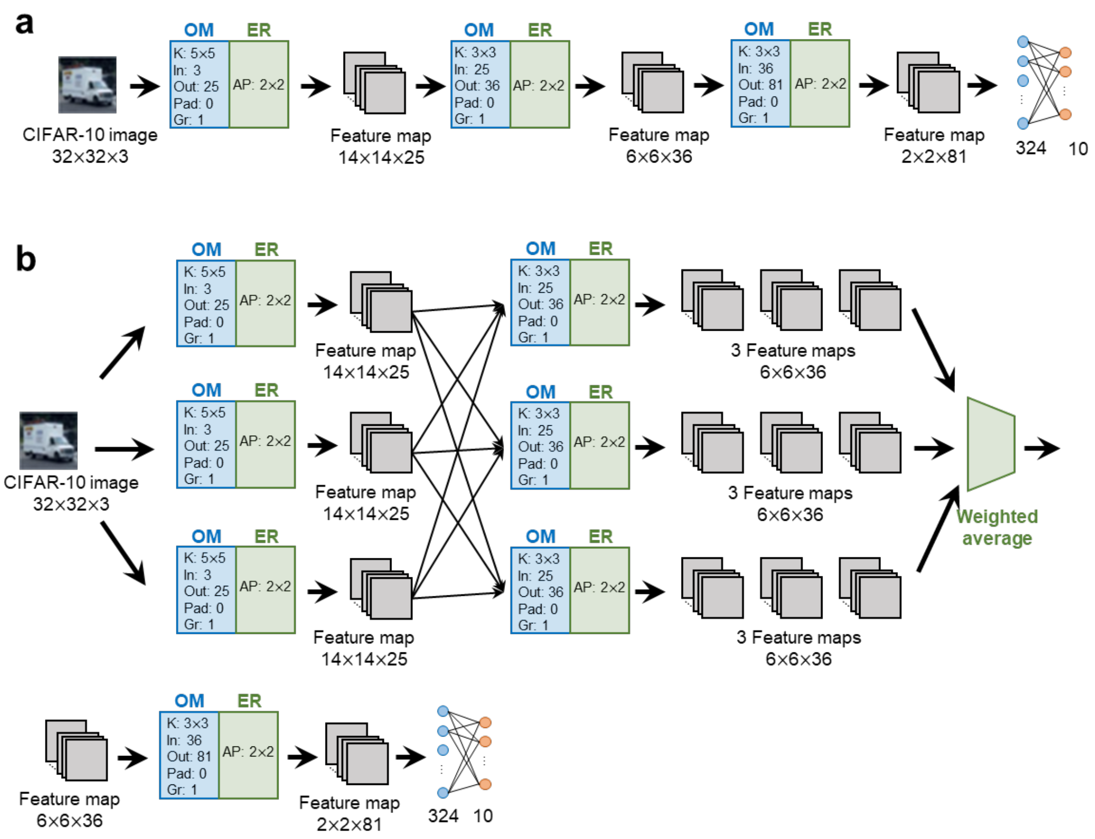

Supplementary Fig. S4 | Detailed network structures (Fig. 2). a, A 3-layer network ONN-3-3. The kernel sizes are 5, 3, 3 for 1st, 2nd, and 3rd optical modulation (OM) layer. A 2x2 average pooling operator is used for the electronic relay layer (ER) of all the 3 layers. The final feature maps are flattened to a 324-d vector and input to a fully-connected layer. **b,** ONN-3-7. Different from ONN-3-3, 3 OMs are used for both the 1st and 2nd layer. The 3 OMs and ERs in the 2nd layer are multiplexed 3 times to generate 9 groups of feature maps. These 9 groups are further weighted averaged and input to the 3rd layer OM and ER. K, kernel size. In, number of input channels. Out, number of output channels. Pad, padding of this convolution layer. Gr, kernel groups. AP, average pooling.

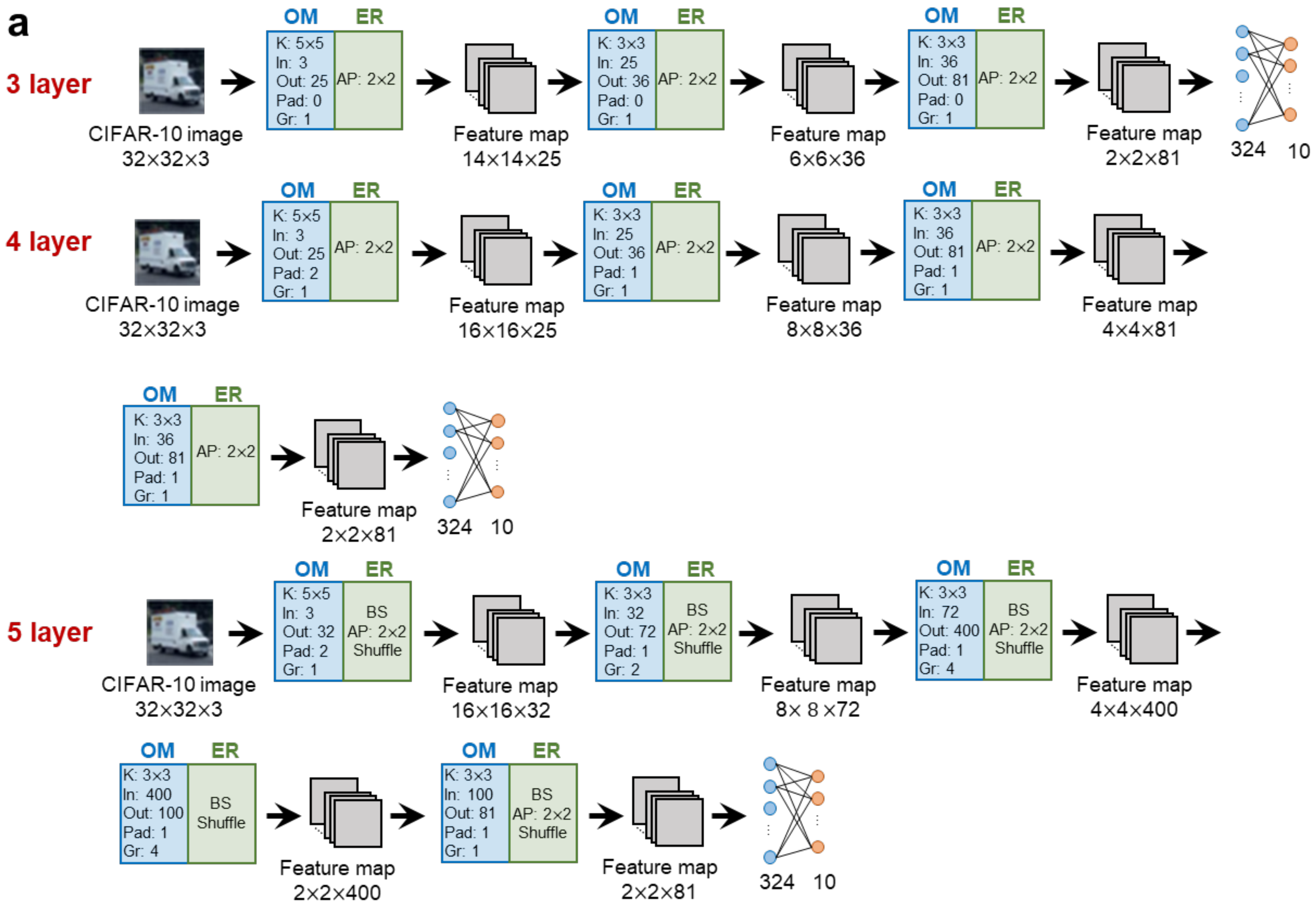
a
3 layer
CIFAR-10 image
32×32×3
OM
ER
K: 5×5
In: 3
Out: 25
Pad: 0
Gr: 1
AP: 2×2
Feature map
14×14×25
OM
ER
K: 3×3
In: 25
Out: 36
Pad: 0
Gr: 1
AP: 2×2
Feature map
6×6×36
OM
ER
K: 3×3
In: 36
Out: 81
Pad: 0
Gr: 1
AP: 2×2
Feature map
2×2×81
324
10
4 layer
CIFAR-10 image
32×32×3
OM
ER
K: 5×5
In: 3
Out: 25
Pad: 2
Gr: 1
AP: 2×2
Feature map
16×16×25
OM
ER
K: 3×3
In: 25
Out: 36
Pad: 1
Gr: 1
AP: 2×2
Feature map
8×8×36
OM
ER
K: 3×3
In: 36
Out: 81
Pad: 1
Gr: 1
AP: 2×2
Feature map
4×4×81
OM
ER
K: 3×3
In: 36
Out: 81
Pad: 1
Gr: 1
AP: 2×2
Feature map
2×2×81
324
10
5 layer
CIFAR-10 image
32×32×3
OM
ER
K: 5×5
In: 3
Out: 32
Pad: 2
Gr: 1
BS
AP: 2×2
Shuffle
Feature map
16×16×32
OM
ER
K: 3×3
In: 32
Out: 72
Pad: 1
Gr: 2
BS
AP: 2×2
Shuffle
Feature map
8× 8 ×72
OM
ER
K: 3×3
In: 72
Out: 400
Pad: 1
Gr: 4
BS
AP: 2×2
Shuffle
Feature map
4×4×400
OM
ER
K: 3×3
In: 400
Out: 100
Pad: 1
Gr: 4
BS
Shuffle
Feature map
2×2×400
OM
ER
K: 3×3
In: 100
Out: 81
Pad: 1
Gr: 1
BS
AP: 2×2
Shuffle
Feature map
2×2×81
324
10

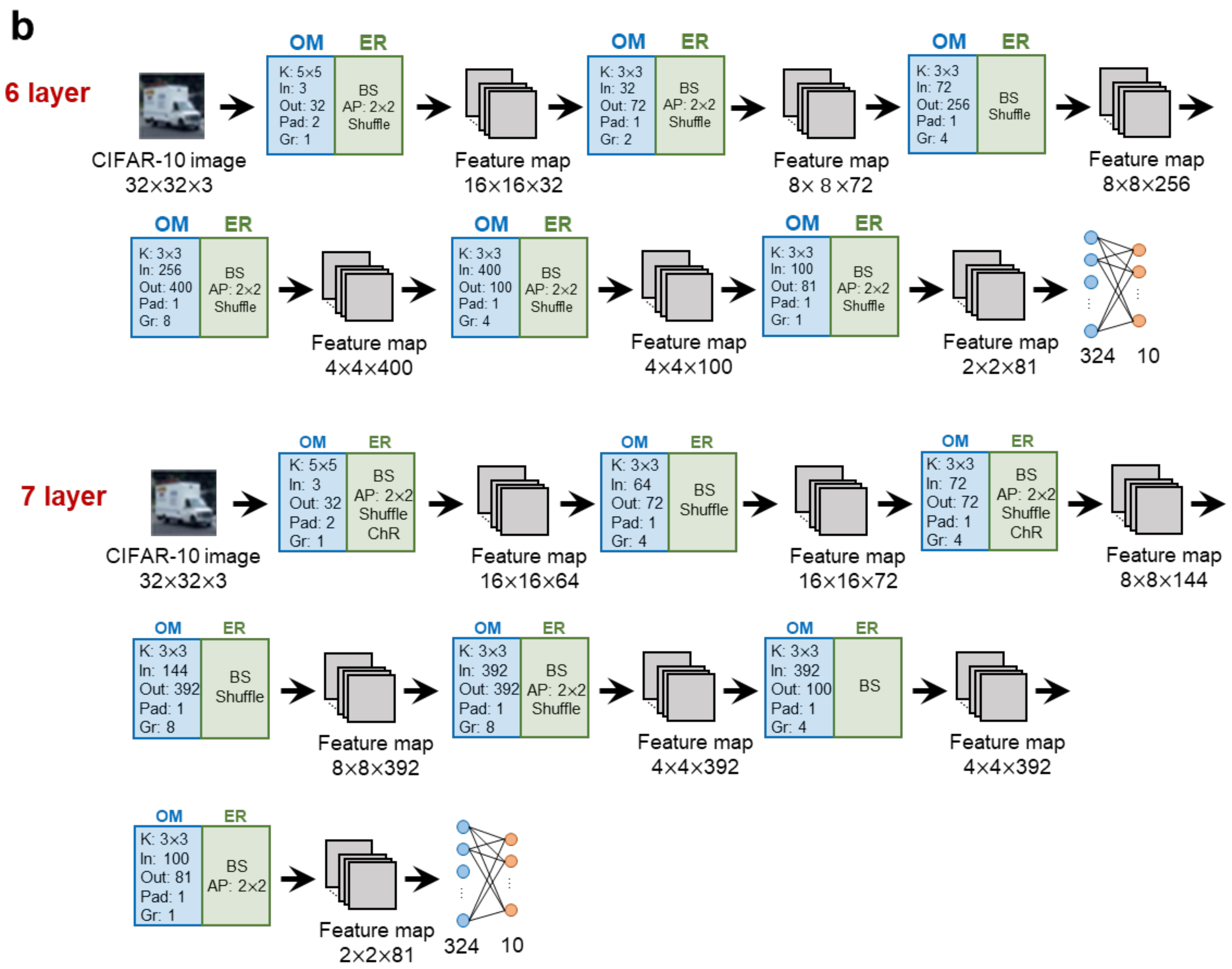
b
6 layer
CIFAR-10 image
32×32×3
OM
ER
K: 5×5
In: 3
Out: 32
Pad: 2
Gr: 1
BS
AP: 2×2
Shuffle
Feature map
16×16×32
K: 3×3
In: 32
Out: 72
Pad: 1
Gr: 2
BS
AP: 2×2
Shuffle
Feature map
8× 8 ×72
K: 3×3
In: 72
Out: 256
Pad: 1
Gr: 4
BS
Shuffle
Feature map
8×8×256
K: 3×3
In: 256
Out: 400
Pad: 1
Gr: 8
BS
AP: 2×2
Shuffle
Feature map
4×4×400
K: 3×3
In: 400
Out: 100
Pad: 1
Gr: 4
BS
AP: 2×2
Shuffle
Feature map
4×4×100
K: 3×3
In: 100
Out: 81
Pad: 1
Gr: 1
BS
AP: 2×2
Shuffle
Feature map
2×2×81
324
10
7 layer
CIFAR-10 image
32×32×3
K: 5×5
In: 3
Out: 32
Pad: 2
Gr: 1
BS
AP: 2×2
Shuffle
ChR
Feature map
16×16×64
K: 3×3
In: 64
Out: 72
Pad: 1
Gr: 4
BS
Shuffle
Feature map
16×16×72
K: 3×3
In: 72
Out: 72
Pad: 1
Gr: 4
BS
AP: 2×2
Shuffle
ChR
Feature map
8×8×144
K: 3×3
In: 144
Out: 392
Pad: 1
Gr: 8
BS
Shuffle
Feature map
8×8×392
K: 3×3
In: 392
Out: 392
Pad: 1
Gr: 8
BS
AP: 2×2
Shuffle
Feature map
4×4×392
K: 3×3
In: 392
Out: 100
Pad: 1
Gr: 4
BS
Feature map
4×4×392
K: 3×3
In: 100
Out: 81
Pad: 1
Gr: 1
BS
AP: 2×2
Feature map
2×2×81
324
10

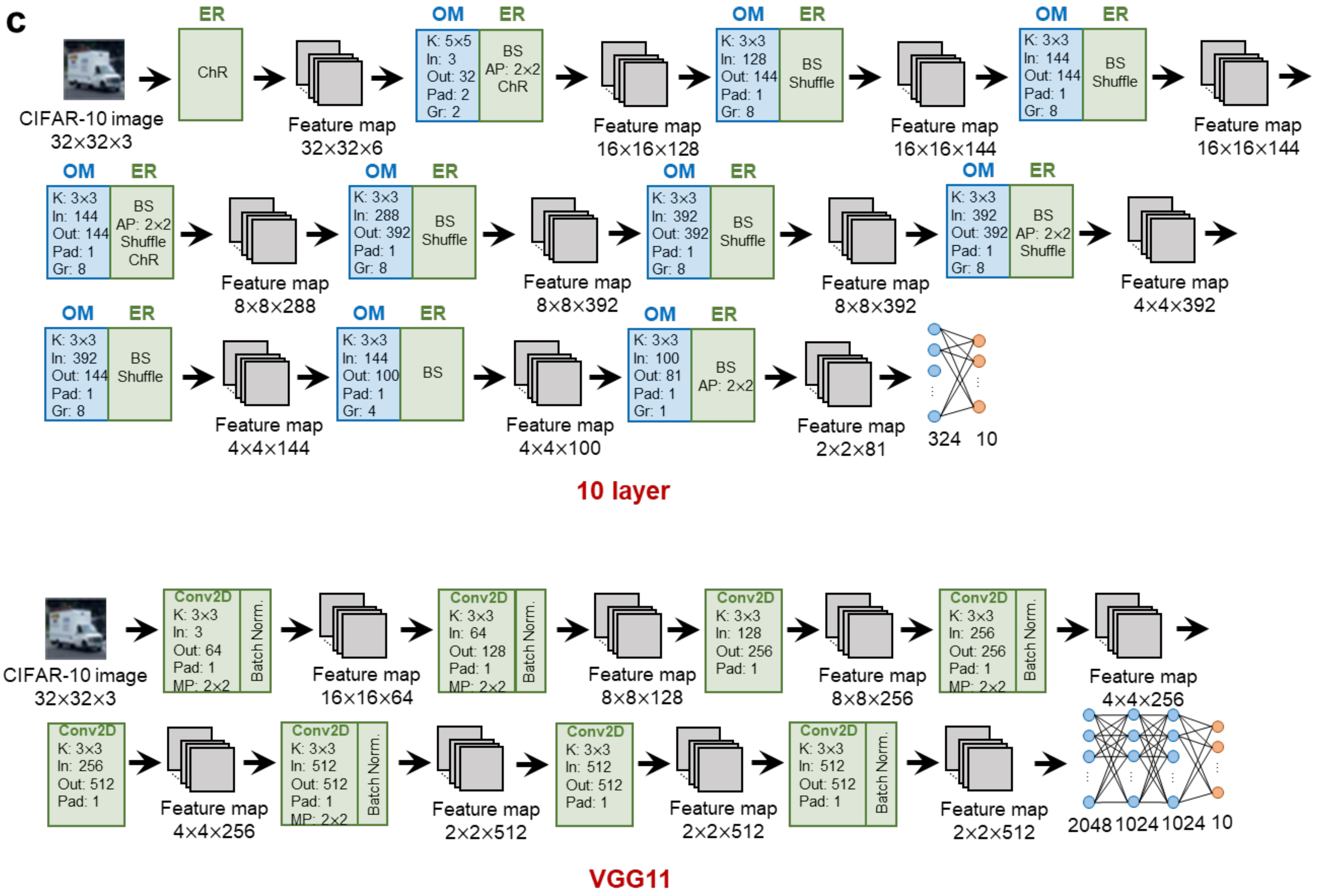

Supplementary Fig. S5 | Detailed network structures (Fig. 3, CIFAR-10). ab, 3-to-7-layer ONN for CIFAR-10 dataset. **c,** The 10-layer ONN and the VGG11 network used for comparison. K, kernel size. In, number of input channels. Out, number of output channels. Pad, padding of this convolution layer. Gr, kernel groups. AP, average pooling. BS, bias and scale operation. Shuffle, channel shuffle operation. ChR, channel repeat operation. Duplicate the original channels. Batch Norm., batch normalization.

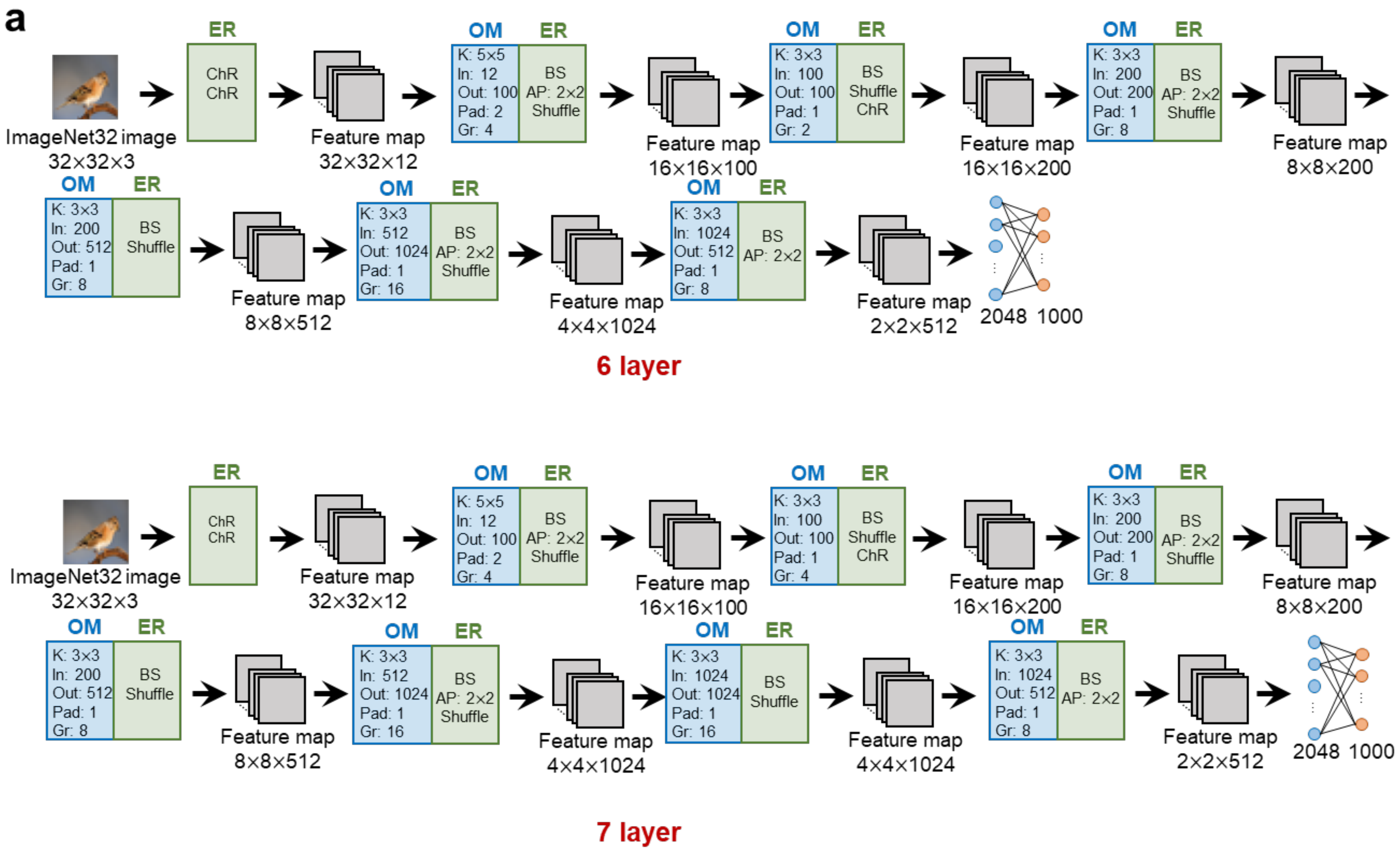
a
ER
ChR
ChR
ImageNet32 image
32×32×3
Feature map
32×32×12
OM
ER
K: 5x5
In: 12
Out: 100
Pad: 2
Gr: 4
BS
AP: 2x2
Shuffle
Feature map
16×16×100
OM
ER
K: 3x3
In: 100
Out: 100
Pad: 1
Gr: 2
BS
Shuffle
ChR
Feature map
16×16×200
OM
ER
K: 3x3
In: 200
Out: 200
Pad: 1
Gr: 8
BS
AP: 2x2
Shuffle
Feature map
8×8×200
OM
ER
K: 3x3
In: 200
Out: 512
Pad: 1
Gr: 8
BS
Shuffle
Feature map
8×8×512
OM
ER
K: 3x3
In: 512
Out: 1024
Pad: 1
Gr: 16
BS
AP: 2x2
Shuffle
Feature map
4×4×1024
OM
ER
K: 3x3
In: 1024
Out: 512
Pad: 1
Gr: 8
BS
AP: 2x2
Feature map
2×2×512
2048 1000
6 layer
ER
ChR
ChR
ImageNet32 image
32×32×3
Feature map
32×32×12
OM
ER
K: 5x5
In: 12
Out: 100
Pad: 2
Gr: 4
BS
AP: 2x2
Shuffle
Feature map
16×16×100
OM
ER
K: 3x3
In: 100
Out: 100
Pad: 1
Gr: 4
BS
Shuffle
ChR
Feature map
16×16×200
OM
ER
K: 3x3
In: 200
Out: 200
Pad: 1
Gr: 8
BS
AP: 2x2
Shuffle
Feature map
8×8×200
OM
ER
K: 3x3
In: 200
Out: 512
Pad: 1
Gr: 8
BS
Shuffle
Feature map
8×8×512
OM
ER
K: 3x3
In: 512
Out: 1024
Pad: 1
Gr: 16
BS
AP: 2x2
Shuffle
Feature map
4×4×1024
OM
ER
K: 3x3
In: 1024
Out: 1024
Pad: 1
Gr: 16
BS
Shuffle
Feature map
4×4×1024
OM
ER
K: 3x3
In: 1024
Out: 512
Pad: 1
Gr: 8
BS
AP: 2x2
Feature map
2×2×512
2048 1000
7 layer

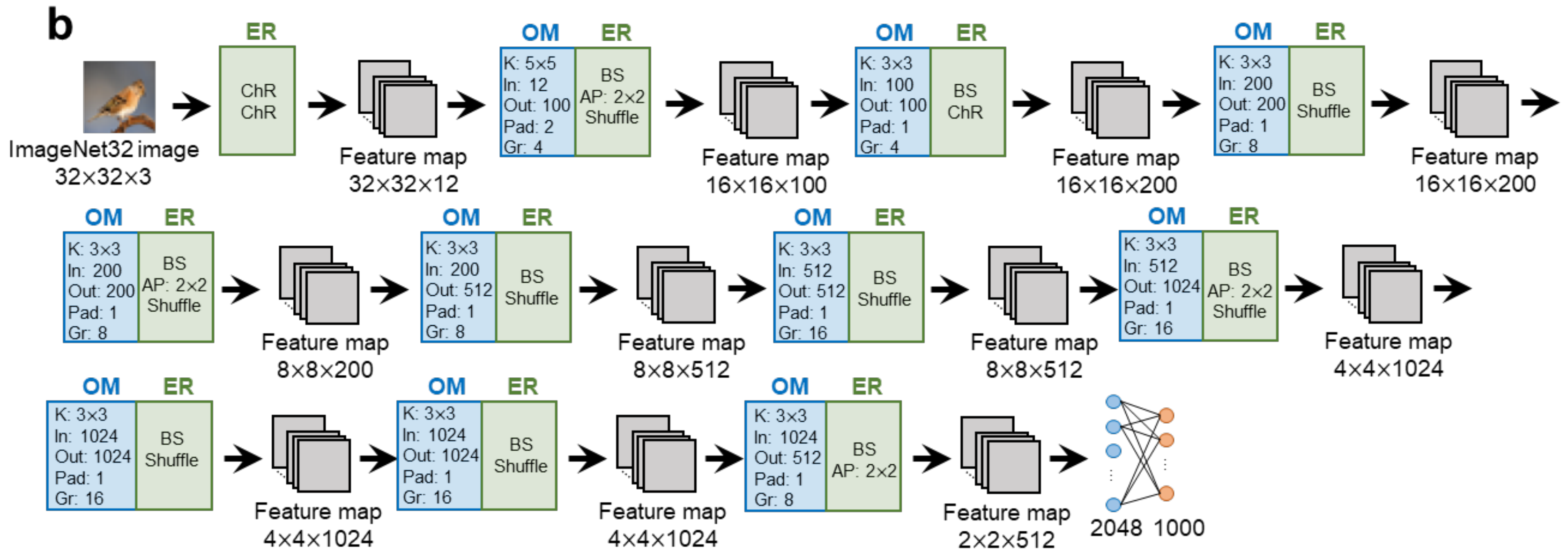
b
ImageNet32 image
32×32×3
ER
ChR
ChR
Feature map
32×32×12
OM
K: 5x5
In: 12
Out: 100
Pad: 2
Gr: 4
ER
BS
AP: 2x2
Shuffle
Feature map
16×16×100
OM
K: 3x3
In: 100
Out: 100
Pad: 1
Gr: 4
ER
BS
ChR
Feature map
16×16×200
OM
K: 3x3
In: 200
Out: 200
Pad: 1
Gr: 8
ER
BS
Shuffle
Feature map
16×16×200
OM
K: 3x3
In: 200
Out: 200
Pad: 1
Gr: 8
ER
BS
AP: 2x2
Shuffle
Feature map
8×8×200
OM
K: 3x3
In: 200
Out: 512
Pad: 1
Gr: 8
ER
BS
Shuffle
Feature map
8×8×512
OM
K: 3x3
In: 512
Out: 512
Pad: 1
Gr: 16
ER
BS
Shuffle
Feature map
8×8×512
OM
K: 3x3
In: 512
Out: 1024
Pad: 1
Gr: 16
ER
BS
AP: 2x2
Shuffle
Feature map
4×4×1024
OM
K: 3x3
In: 1024
Out: 1024
Pad: 1
Gr: 16
ER
BS
Shuffle
Feature map
4×4×1024
OM
K: 3x3
In: 1024
Out: 1024
Pad: 1
Gr: 16
ER
BS
Shuffle
Feature map
4×4×1024
OM
K: 3x3
In: 1024
Out: 512
Pad: 1
Gr: 8
ER
BS
AP: 2x2
Feature map
2×2×512
2048 1000

10 layer

c

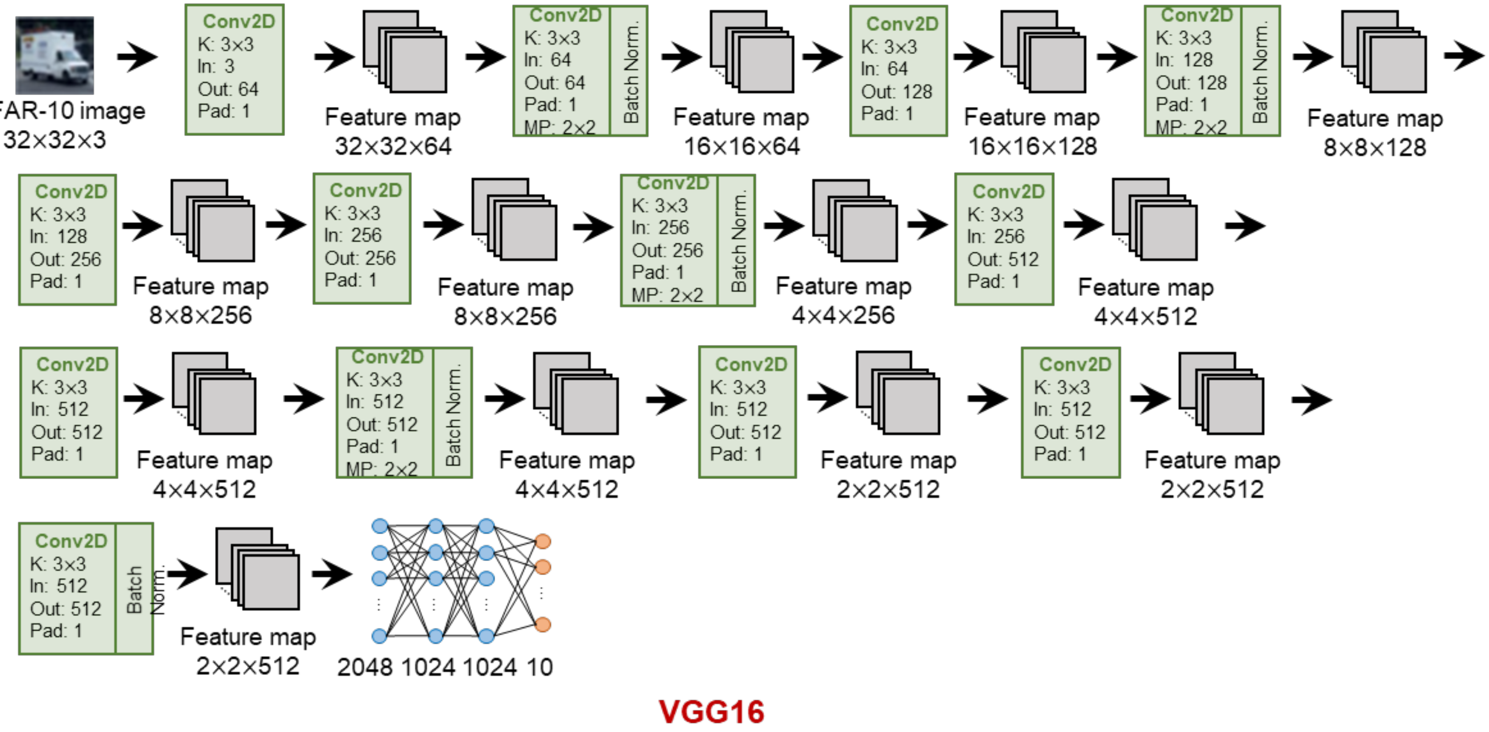

Supplementary Fig. S6 | Detailed network structures (Fig. 3, ImageNet32). ab, 6-layer, 7-layer, and 8-layer ONNs for ImageNet32 dataset. The final feature maps are flattened to a 2048-d vector and input to a 2048-1000 fully-connected layer. **c,** VGG16 network used for comparison. K, kernel size. In, number of input channels. Out, number of output channels. Pad, padding of this convolution layer. Gr, kernel groups. AP, average pooling. BS, bias and scale operation. Shuffle, channel shuffle operation. ChR, channel repeat operation. Duplicate the original channels. Batch Norm., batch normalization.

319

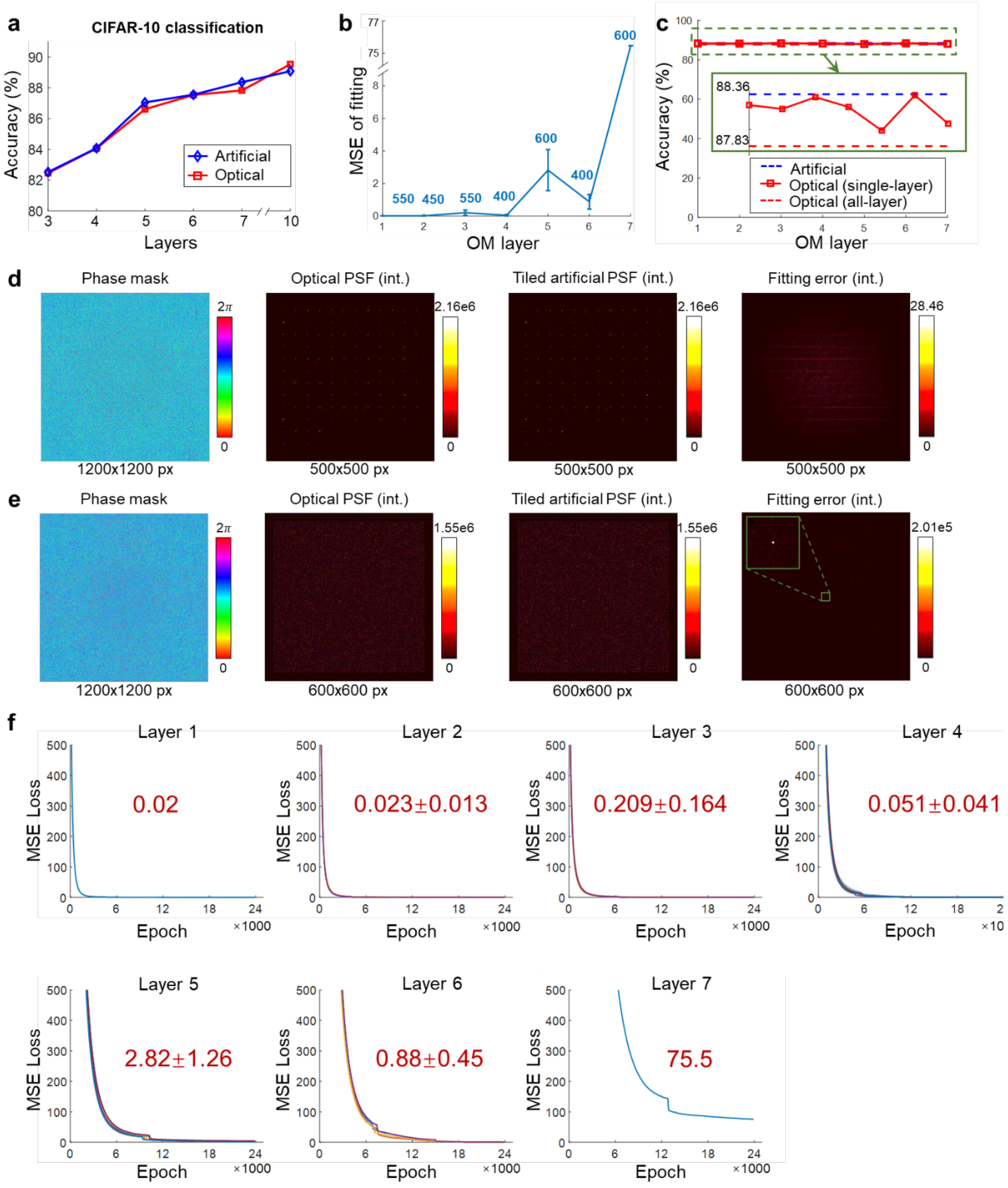

a
CIFAR-10 classification
Accuracy (%)
Layers
Artificial
Optical
b
MSE of fitting
OM layer
550 450 550 400 600 400 600
c
Accuracy (%)
OM layer
88.36
87.83
Artificial
Optical (single-layer)
Optical (all-layer)
d
Phase mask
Optical PSF (int.)
Tiled artificial PSF (int.)
Fitting error (int.)
2π
2.16e6
28.46
1200x1200 px
500x500 px
e
Phase mask
Optical PSF (int.)
Tiled artificial PSF (int.)
Fitting error (int.)
1.55e6
2.01e5
1200x1200 px
600x600 px
f
Layer 1
0.02
Layer 2
0.023±0.013
Layer 3
0.209±0.164
Layer 4
0.051±0.041
Layer 5
2.82±1.26
Layer 6
0.88±0.45
Layer 7
75.5
MSE Loss
Epoch
×1000

Supplementary Fig. S7 | Optical-artificial fitting error. a, The classification accuracies of the ONNs at the artificial-neuron level and the optical-neuron level. The number of layers contains the last fully-connected (FC) layer, so the 3-layer ONN consists of 3 OM layers. The average accuracy difference is 0.25$\pm$0.24%, without finetuning the FC layer using the optical outputs. Interestingly, the optical-neuron level accuracy may outperform the artificial-neuron level, possibly because the smooth constraint inside the optical neurons reduces the noise of the input images. **b**, The 7-layer ONN is taken as an example, and detailly analyzed in b and c. Subfig. b plots the fitting error (MSE) of the optical neurons in the 7 OM layers. The error bars denote the standard deviation computed from the multiple phase masks in the same layer. The numbers above the curve denote the size of the region to fit (PSF size). We can see that the layers with larger PSF are more difficult to fit, as the phase mask has to be trained to fit more pixels. Also, the deeper layers (e.g. the last layer) are difficult to fit, as the deeper layers have smaller input sizes and more kernels, leading to more high-frequency components in the PSF. **c**, The accuracies after fitting a single layer. The layer with higher fitting error also results in a larger performance drop. Although the fitting error of the last OM layer is much larger than the other layers, the cause performance drop is smaller than the 5th layer. **de**, The trained phase and PSF of the 2nd and 7th layer (only 1 phase mask in these two layers). From left to right, the trained phase mask, the PSF (impulse) of the fitted optical neuron, the tiled PSF of the artificial neuron, and the difference between the two PSFs. The two PSFs and the differences are all complex matrices, we only show the intensity (absolute square) here. The maximum difference appears in the center of the PSF, and the errors of the remaining pixels are much smaller, especially for the 7th layer PSF. Thus, although the fitting error of the 7th layer seems large, the performance drop is acceptable. **f**, The convergence curves of the optical neurons of the 7-layer ONN. The optical neurons of the same layer are drawn in the same subfigure. The red numbers on the subfigures denote the MSE loss after convergence.

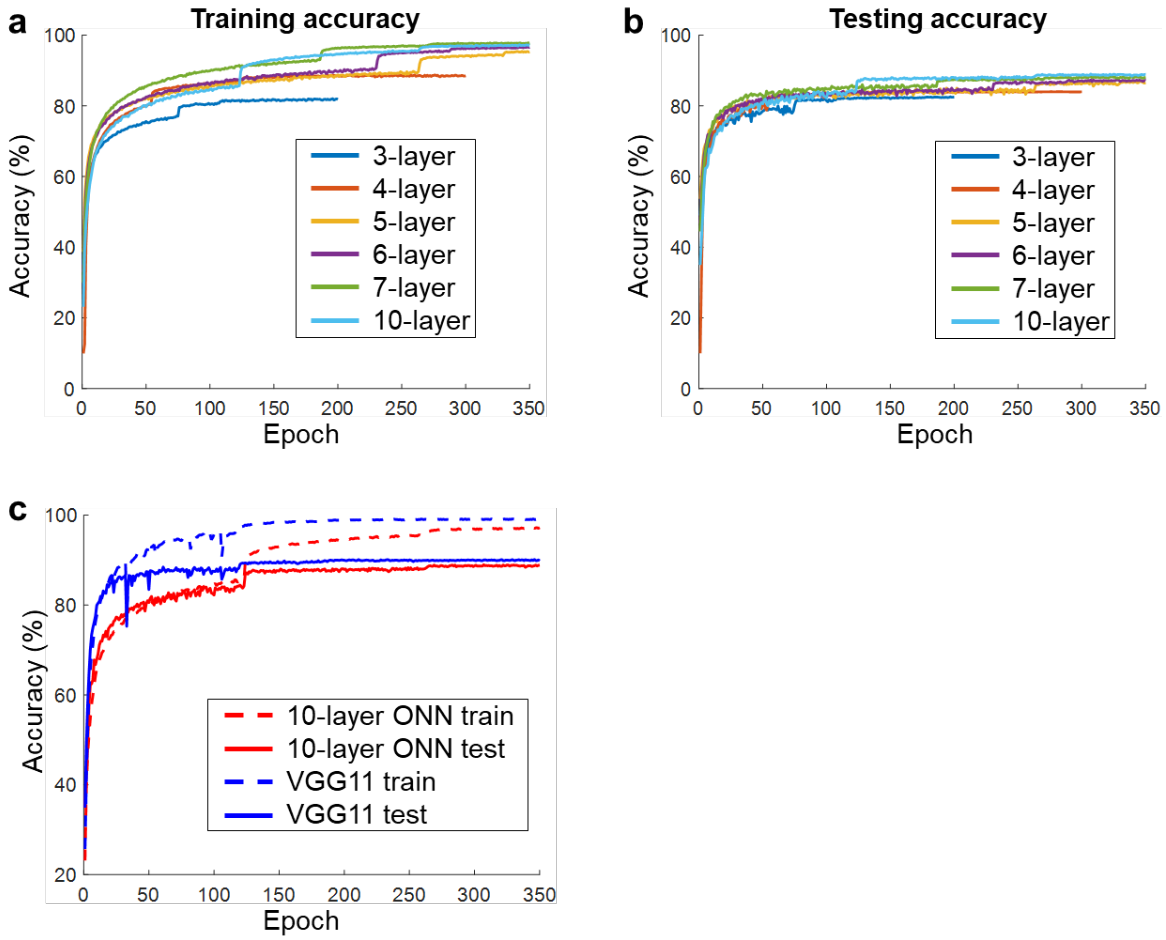

Supplementary Fig. S8 | Convergence analysis of DANTE. a, The training accuracy curve of the 3- to 10- layer ONN on the CIFAR-10 dataset. (Fig. 3c). **b**, The testing accuracy curve of the 3- to 10- layer ONN on the CIFAR-10 dataset. (Fig. 3c). The testing convergence rates of different layer ONNs are similar, but the deeper networks achieve higher performance after convergence. Adam optimizer with ReduceLROnPlateau (reduce learning rate when a metric has stopped improving) learning-rate scheduler is used to optimize the parameters. The initial learning rate is set to 0.05, and the patience and factor of the scheduler are set to 30 and 0.2, respectively. **c**, The training and testing accuracy curve of the 10-layer ONN and the VGG11 network. These two networks achieve similar performance after convergence, but the VGG11 network converges faster. One of the possible reasons is that we have to use the Sigmoid function to normalize the intensity field for phase conversion, while the VGG11 network uses ReLU activation functions.

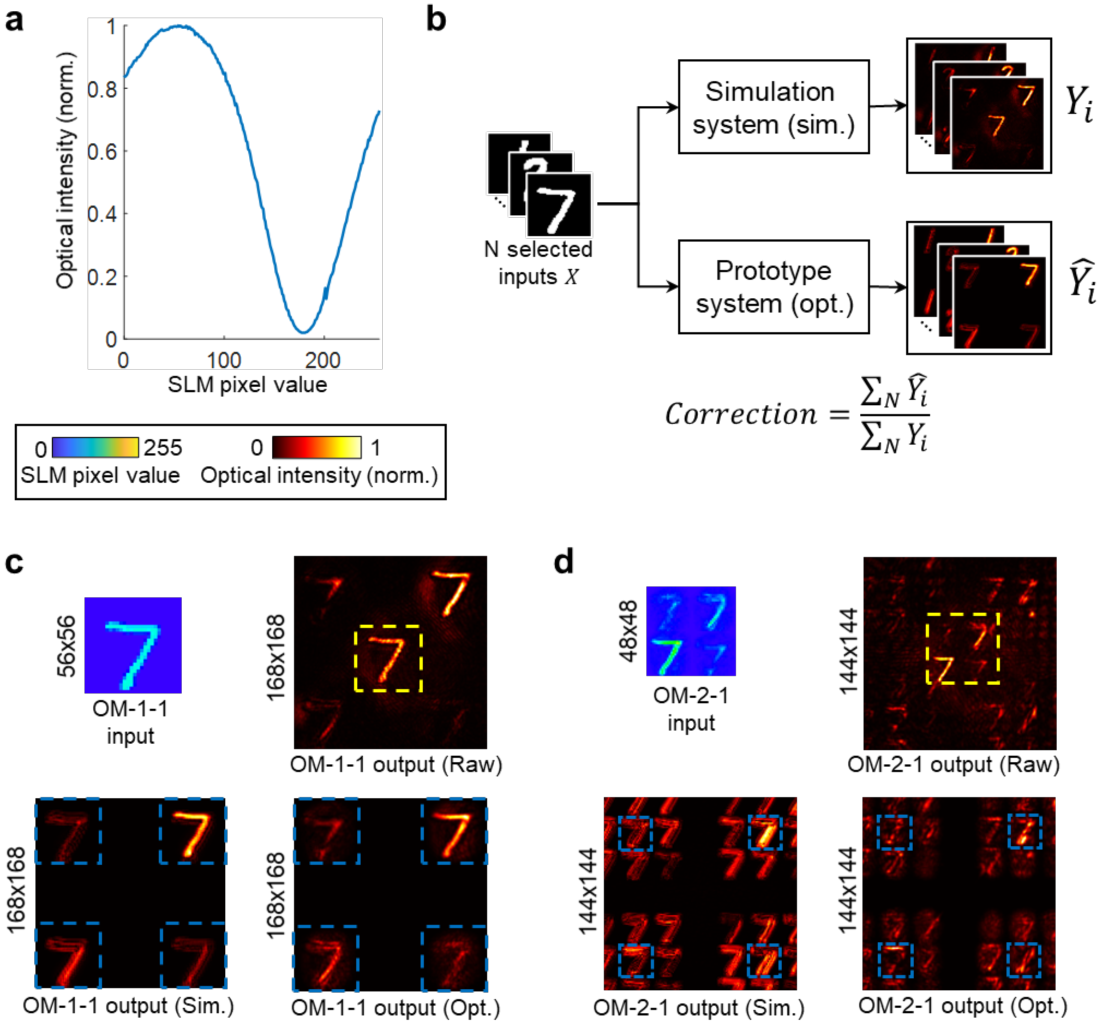

Supplementary Fig. S9 | DANTE on the custom ONN system. a, Calibrated amplitude modulation curve of SLM. The extinction ratio is around 50 (optical intensity). The curve is fitted using a differentiable cosine function and added to the neural network model. **b,** Method to compute the intensity correction mask. N selected inputs X are input to the simulation system and prototype system to compute the simulation results and optical results. The intensity correction map can then be computed from the summed intensity of these two results. The correction map is then applied to all the captured sample. **c,** Raw output intensity map (raw), corrected intensity map (opt.), and simulated intensity map of the first OM layer of the MNIST network. Due to the imperfect modulation efficiency (< 100%) of the SLM and the assembly error of the physical system, there will be a strong zero-order diffraction pattern in the center of the raw output intensity map (yellow dash box). After correction, the zero-order diffraction is removed. Besides, the system noise is also suppressed. In network implementation, we only need to correct the pixel regions that will be used in the next layer (4 blue dash boxes). **d,** Raw output intensity map (raw), corrected intensity map (opt.), and simulated intensity map of the second OM layer of the MNIST network. Similarly, an intensity correction mask is used to suppress the zero-order diffraction and noise.

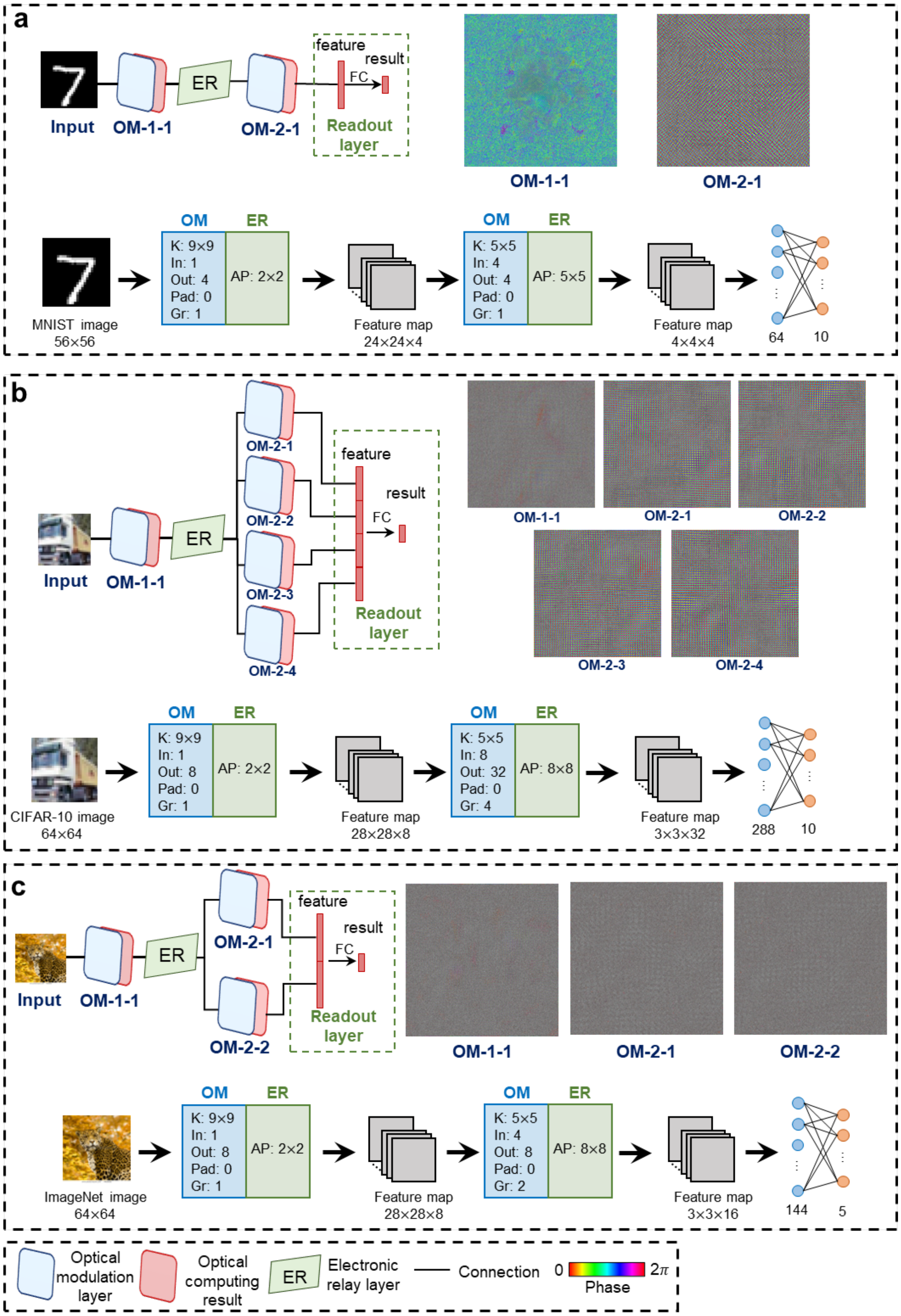

Supplementary Fig. S10 | Network structures and learned optical neuron parameters of the ONN system. a, MNIST network. Top left shows the network structures. Top right shows the learned network parameters (phase mask). Bottom row shows the detailed parameters of the artificial neurons of each OM and ER layers. **b,** CIFAR-10 network. **c,** ImageNet-64 network

Supplementary references

1. Joseph W. Goodman. *Introduction to Fourier Optics.* (Roberts and Company Publishers, 2005).
2. Coherent optical imaging — Advanced Optical Imaging. https://qiweb.tudelft.nl/aoi/coherentimaging/coherentimaging.html#object-and-image-distance-equal-to-focal-distance.
3. Ding, X., Zhang, X., Han, J. & Ding, G. Scaling Up Your Kernels to 31×31: Revisiting Large Kernel Design in CNNs. in *IEEE/CVF Conference on Computer Vision and Pattern Recognition* (2022). doi:10.1109/CVPR52688.2022.01166.
4. Simonyan, K. & Zisserman, A. Very deep convolutional networks for large-scale image recognition. in *International Conference on Learning Representations* (2015).
5. He, K., Zhang, X., Ren, S. & Sun, J. Deep residual learning for image recognition. in *IEEE Conference on Computer Vision and Pattern Recognition* (2016). doi:10.1109/CVPR.2016.90.
6. Krizhevsky, A., Sutskever, I. & Hinton, G. E. ImageNet Classification with Deep Convolutional Neural Networks. *Adv Neural Inf Process Syst* **25**, (2012).
7. Szegedy, C., Vanhoucke, V., Ioffe, S., Shlens, J. & Wojna, Z. Rethinking the Inception Architecture for Computer Vision. in *IEEE Conference on Computer Vision and Pattern Recognition* (2016). doi:10.1109/CVPR.2016.308.
8. FFT Benchmark Methodology. https://www.fftw.org/speed/method.html.
9. Dalir, H. *et al.* Massively parallel amplitude-only Fourier neural network. *Optica* **7**, 1812–1819 (2020).
10. Zhou, T. *et al.* Large-scale neuromorphic optoelectronic computing with a reconfigurable diffractive processing unit. *Nat Photonics* **15**, 367–373 (2021).
11. Zagoruyko, S. & Komodakis, N. Wide Residual Networks. in *British Machine Vision Conference* (2016).
12. Dou, H. *et al.* Residual D^2NN: training diffractive deep neural networks via learnable light shortcuts. *Opt Lett* **45**, 2688–2691 (2020).
13. Rahman, M. S. S., Li, J., Mengu, D., Rivenson, Y. & Ozcan, A. Ensemble learning of diffractive optical networks. *Light Sci Appl* **10**, 1–13 (2021).
14. Lin, X. *et al.* All-optical machine learning using diffractive deep neural networks. *Science (1979)* **361**, 1004–1008 (2018).
15. Yan, T. *et al.* Fourier-space Diffractive Deep Neural Network. *Phys Rev Lett* **123**, 023901 (2019).
16. Chen, H. *et al.* Diffractive Deep Neural Networks at Visible Wavelengths. *Engineering* **7**, 1483–1491 (2021).
17. Liu, C. *et al.* A programmable diffractive deep neural network based on a digital-coding metasurface array. *Nat Electron* **5**, 113–122 (2022).
18. Li, Y. *et al.* Multiscale diffractive U-Net: a robust all-optical deep learning framework modeled with sampling and skip connections. *Opt Express* **30**, 36700–36710 (2022).
