# Supplementary material for: Training large-scale optoelectronic neural networks with dual-neuron optical-artificial learning
Source: Nat Commun. 2023 Nov 4;14:7110. doi: 10.1038/s41467-023-42984-y (PMC10625607; doi:10.1038/s41467-023-42984-y)
Supplement: Supplementary file 3 — Description of Additional Supplementary Files [file 41467_2023_42984_MOESM3_ESM.pdf]

Description of Additional Supplementary Files

File name: Supplementary Movie 1

Description: Results of our physical ONN system on the MNIST dataset.

File name: Supplementary Movie 2

Description: Results of our physical ONN system on the ImageNet-32 dataset.
